# KLF11 regulates lung adenocarcinoma ferroptosis and chemosensitivity by suppressing GPX4

**DOI:** 10.1038/s42003-023-04959-z

**Published:** 2023-05-29

**Authors:** Guangyin Zhao, Jiaqi Liang, Guangyao Shan, Jie Gu, Fengkai Xu, Chunlai Lu, Teng Ma, Guoshu Bi, Cheng Zhan, Di Ge

**Affiliations:** Department of Thoracic Surgery, Zhongshan Hospital, Fudan University, Shanghai, China

**Keywords:** Lung cancer, Oncology

## Abstract

Ferroptosis, an iron-dependent non-apoptotic cell death, has been shown to play a vital role in tumor proliferation and chemotherapy resistance. Here, we report that KLF11 inhibits lung adenocarcinoma (LUAD) cell proliferation and promotes chemotherapy sensitivity by participating in the GPX4-related ferroptosis pathway. Through an RNA-sequence screen from LUAD cells pretreatment with ferroptosis inducers (FINs), we discovered that KLF11 expression was significantly higher in FINs-treated cells, suggesting that KLF11 may be involved in ferroptosis. Overexpression of KLF11 promoted LUAD cells to undergo ferroptosis alterations. Meanwhile, upregulation of KLF11 expression also inhibited cell proliferation and increased chemosensitivity, whereas knockout of KLF11 did the opposite. With ChIP-Seq and RNA-Seq, we identified GPX4 as a downstream target of KLF11. Through ChIP-qPCR and dual luciferase assay, we clarified that KLF11 binds to the promoter region of GPX4 and represses its transcription. Restored GPX4 expression antagonized the ability of KLF11 to promote ferroptosis, increase chemotherapy sensitivity and inhibit cell proliferation in vitro and in vivo. Clinically, KLF11 declined in LUAD and its low expression was associated with reduced patient survival. Our findings established the function of KLF11 to promote ferroptosis in LUAD, thereby inhibiting cell proliferation and enhancing the efficacy of chemotherapy.

## Abstract (web summary)

KLF11 inhibits lung adenocarcinoma cell proliferation and promotes chemotherapy sensitivity by repressing transcription of GPX4 and promoting ferroptosis.

## Introduction

Lung adenocarcinoma (LUAD) is the most common subtype of lung cancer which is the leading cause of cancer death worldwide^1,2^. LUAD has a relatively strong proliferative capacity with a poor survival outcome, and chemotherapy remains one of the main treatments for this type of malignant tumor. However, resistance to chemotherapy greatly affects the prognosis of patients with LUAD^3–5^. Therefore, it is imperative to explore ways to inhibit LUAD proliferation and increase the sensitivity of chemotherapy in LUAD.

Ferroptosis, an iron-dependent form of regulatory cell death caused by excessive lipid peroxidation, is associated with a variety of biological contexts, from development to aging, immunity, and carcinogenesis, while loss of ferroptosis can drive tumorigenesis and promote tumor proliferation^6^. Importantly, ferroptosis has been proven to be implicated in resistance to chemotherapy, and induction of ferroptosis could enhance chemotherapy sensitivity^7^. Glutathione peroxidase 4 (GPX4) is a central suppressor of the ferroptosis process, reducing lipid peroxidation by decreasing the conversion of GSH, thereby inhibiting ferroptosis^8,9^. Moreover, studies have found that increased GPX4 expression is closely associated with tumorigenesis and metastasis^10^. However, the process that affects ferroptosis by regulating GPX4 in cancer remains unclear.

Kruppel-like factor 11 (KLF11) is a member of the Kruppel-like zinc finger transcription factor family. In this study, we found KLF11 was up-regulated in LUAD cell lines after the FINs treatment, indicating that KLF11 may be involved in the regulation of ferroptosis in LUAD. However, the involvement of KLF11 in ferroptosis and the mechanism have not been reported. Here, with the combination of transcriptome sequencing and ChIP-seq, we systematically identified KLF11 as a transcription repressor for GPX4 and revealed that overexpression of KLF11 stimulates the ferroptosis of LUAD, thus inhibiting cell growth and sensitizing LUAD cells to chemotherapy.

## Results

### KLF11 involves ferroptosis in LUAD

**Fig. 1:**
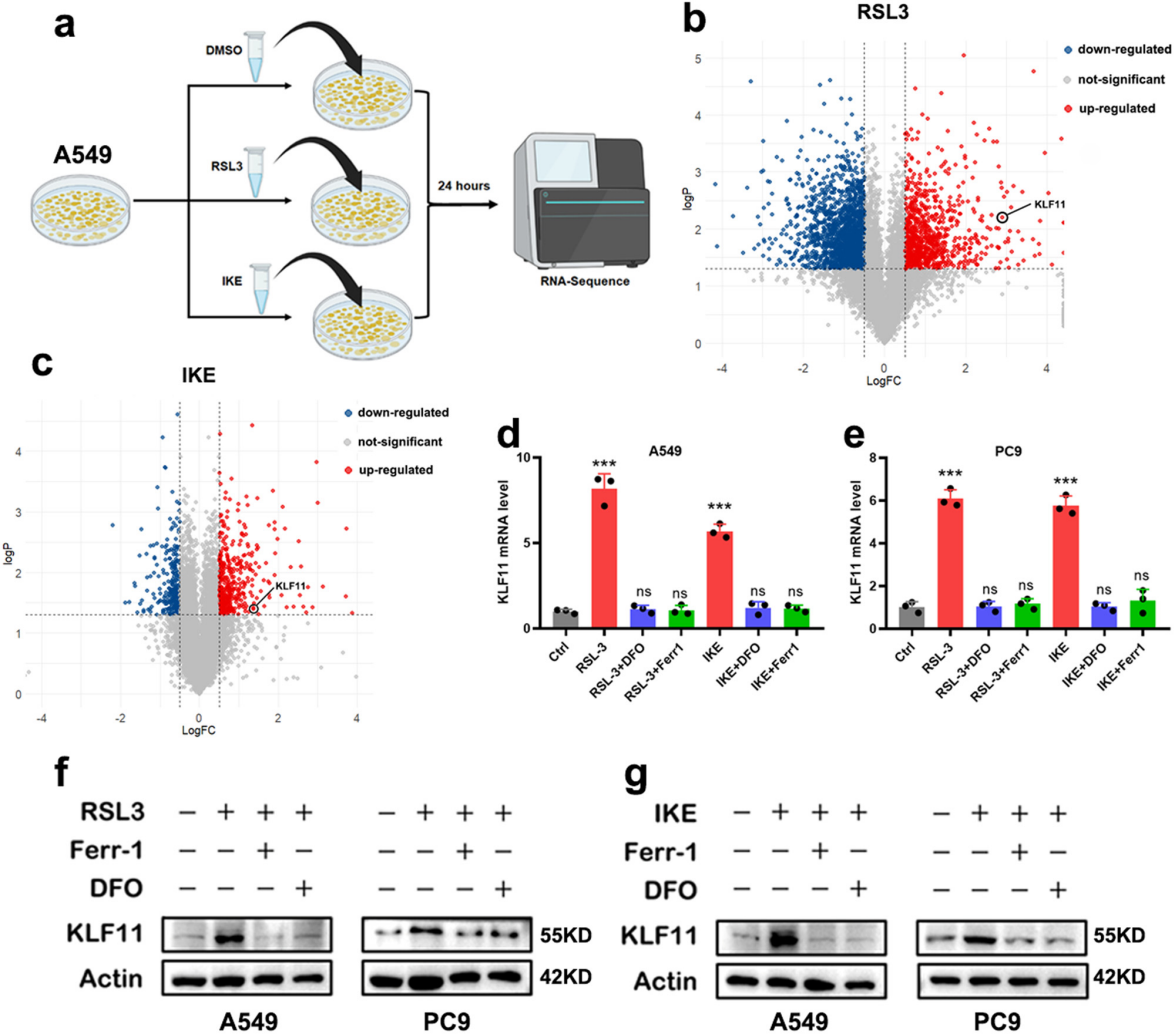
Identification of KLF11 as involved in ferroptosis in LUAD. **a** Schematic diagram of RNA-seq screening workflow in A549 cell line. **b**, **c** Volcano plot illustrating KLF11 involved in regulating ferroptosis induced by RSL3 and IKE. **d**, **e** KLF11 RNA level was measured after treatment with RSL3 or DFO + RSL3 or Ferr1 + RSL3 in A549 and PC9 cell lines. **f**, **g** KLF11 protein level was measured after treatment with RSL3 or DFO + RSL3 or Ferr1 + RSL3 in A549 and PC9 cell lines.

Firstly, we induced ferroptosis using FINs (RSL3 and IKE) in the A549 lung cancer cells and RNA sequencing was performed on treated and non-treated cells (Fig. 1a). We found the mRNA level of KLF11 was significantly upregulated in RSL3 or IKE-treated A549 cell lines compared with the non-treated group (Fig. 1b, c). To corroborate the sequencing results, qPCR and western blot were then used to detect the KLF11 RNA and protein expression under different treatments in LUAD cells. Consistent with RNA-Seq results, KLF11 mRNA was upregulated after the addition of FINs in A549 and PC9 cells, while no significant change in KLF11 expression was observed after the simultaneous addition of ferroptosis-inhibitor (Ferr-1 and DFO, the potent and selective inhibitor of ferroptosis) compared with the control group (Fig. 1d, e). At the protein level, consistent results were obtained (Fig. 1f, g), indicating that ferroptosis activates KLF11’s expression and KLF11 may participate in the process of ferroptosis.

### KLF11 significantly affects the sensitivity of LUAD cells to ferroptosis, proliferation ability, and sensitivity to chemotherapy

**Fig. 2:**
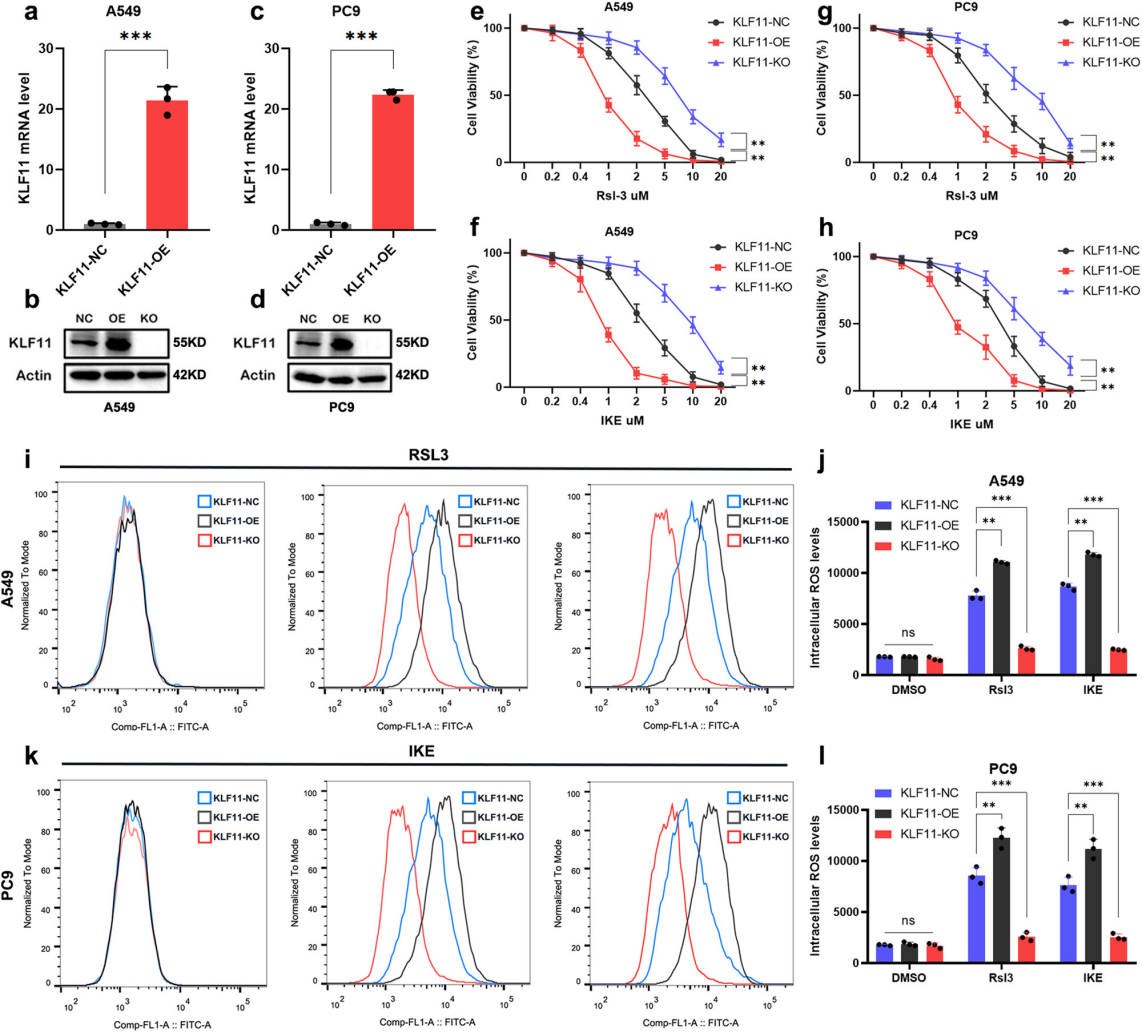
Dysregulated KLF11 expression affects the cytotoxicity and intracellular ROS levels of LUAD cell lines induced by RSL3 and IKE-induced. **a**–**d** qRT-PCR and western blot confirming the overexpression and knock-out of KLF11 in A549 and PC9 cells. **e**–**h** CCK-8 assay to detect the cell activity of A549 and PC9 cells with different levels of KLF11 after 24 h of treatment with different doses of RSL3 and IKE. **i**–**l** Lipid peroxidation was measured by flow cytometry after C11-BODIPY staining in KLF11-NC, KLF11-OE, and KLF11-KO treatment by RSL3 or IKE.

**Fig. 3:**
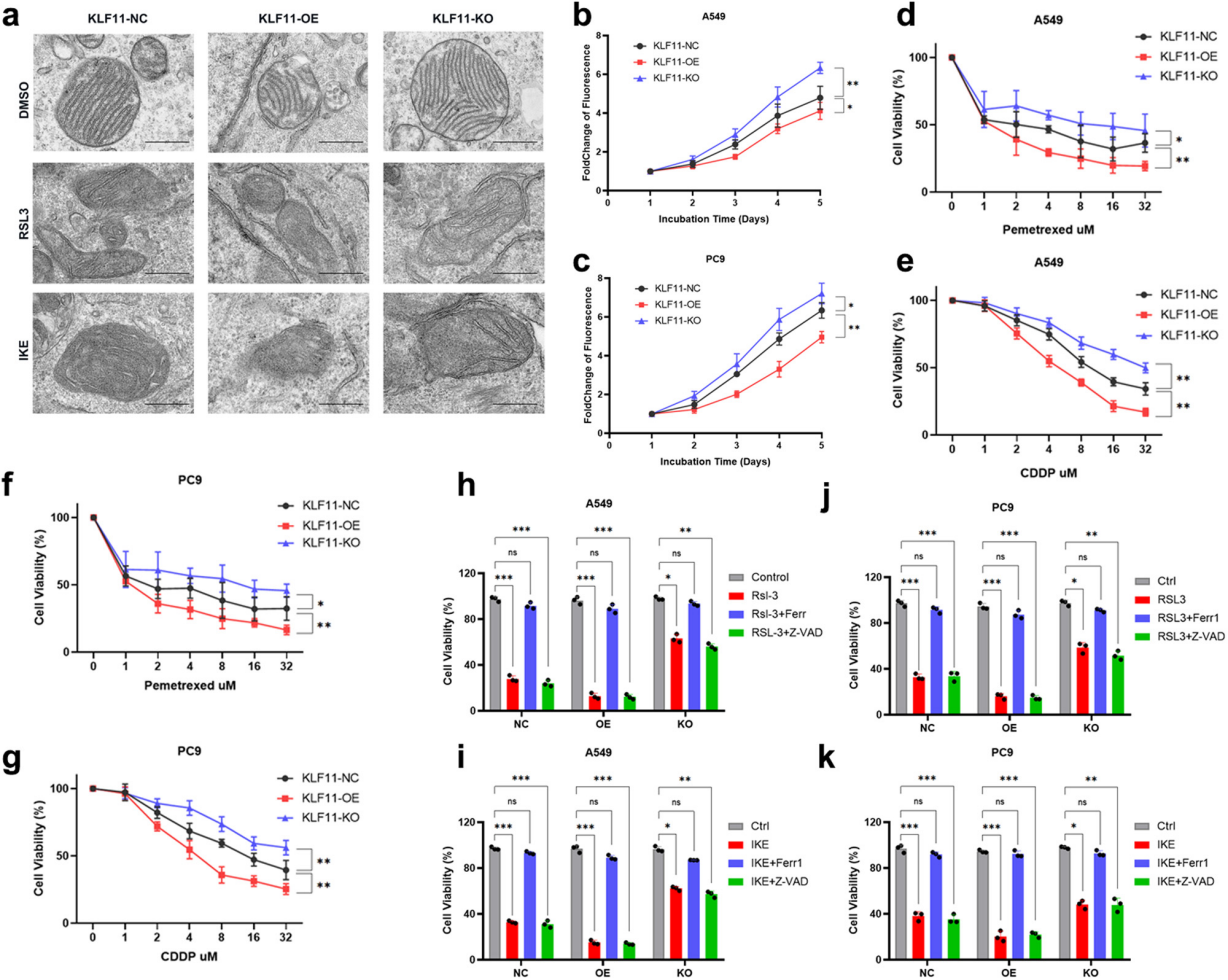
Dysregulation of KLF11 expression causes abnormal mitochondrial morphology in RSL3 or IKE-treated LUAD cells and affects the proliferation ability and sensitivity to chemotherapeutic agents. **a** Characteristic alterations of mitochondria in A549 cells treated with RSL3 or IKE analyzed by TEM, Scale bars: 50 nm. **b**, **c** Dysregulation of KLF11 expression affects the proliferation ability of A549 and PC9 cells. **d**–**g** Dose-toxicity curves showing the viability of A549 and PC9 cells transfected with KLF11-NC, KLF11-OE, and KLF11-KO upon CDDP or Pem treatment at the indicated concentrations for 48 h. **h**–**k** The viability of A549 and PC9 cells transfected with KLF11-NC, KLF11-OE, and KLF11-KO were measured after 24 h treatment with RSL3/IKE, RSL3/IKE+Ferr1, or RSL3/IKE+Z-VAD.

We next found that KLF11 was significantly less expressed in LUAD cell lines than in normal bronchial epithelial cells (Supplementary Fig. 1a). Given the above results, we used two LUAD cell lines, A549 and PC9, to generate KLF11-overexpression(OE) and KLF11-knockout (KO) cells with the CRISPR/Cas9 tool. Against the negative control (NC) group, qRT-PCR and western blot showed that KLF11 was successfully overexpressed or knocked out (Fig. 2a–d). After RSL3 or IKE treatment, the activity of A549 and PC9 cells in the KLF11-OE group was significantly decreased and the intracellular lipid peroxidation level was increased (Fig. 2e–l). In contrast, the knockout of KLF11 resulted in a significant increase in cellular activities and a significant decrease in intracellular lipid peroxidation levels compared with the KLF11-NC group (Fig. 2e–l). As is known, a characteristic change in cells undergoing ferroptosis is the reduction in mitochondrial volume, the condensed mitochondrial membrane densities, the reduction or disappearance of mitochondrial cristae, and the rupture of the outer mitochondrial membrane^11^. We next conduct Transmission electron microscopy (TEM) analysis for further study. After induced by FINs, KLF11-OE A549 cells contained more shrunken mitochondria with elevated membrane density and reduction of mitochondrial cristae compared to KLF11-NC and KO group (Fig. 3a). We subsequently explored the effect of KLF11 on the proliferative capacity of LUAD cells using the CCK-8 assay and found that overexpression of KLF11 significantly inhibited the proliferation ability of A549 and PC9 cells, while knockout of KLF11 obtained the opposite result (Fig. 3b, c). Furthermore, KLF11 was found to enhance the toxic effects of chemotherapeutic agents (CDDP and Pem) on LUAD cells (Fig. 3d–g). Subsequently, KLF11-promoted ferroptosis in LUAD cells can be rescued by ferroptosis inhibitor Ferrostatin-1(Ferr-1), but not the apoptosis inhibitor Z-VAD-FAM (Fig. 3h–k). Taken together, the results suggest that KLF11 promotes FINs-induced ferroptosis, inhibits cell proliferation, and enhances the chemotherapeutic agent’s responsibility in LUAD.

### Overexpression of KLF11 promotes cellular ferroptosis by decreasing GPX4 expression

**Fig. 4:**
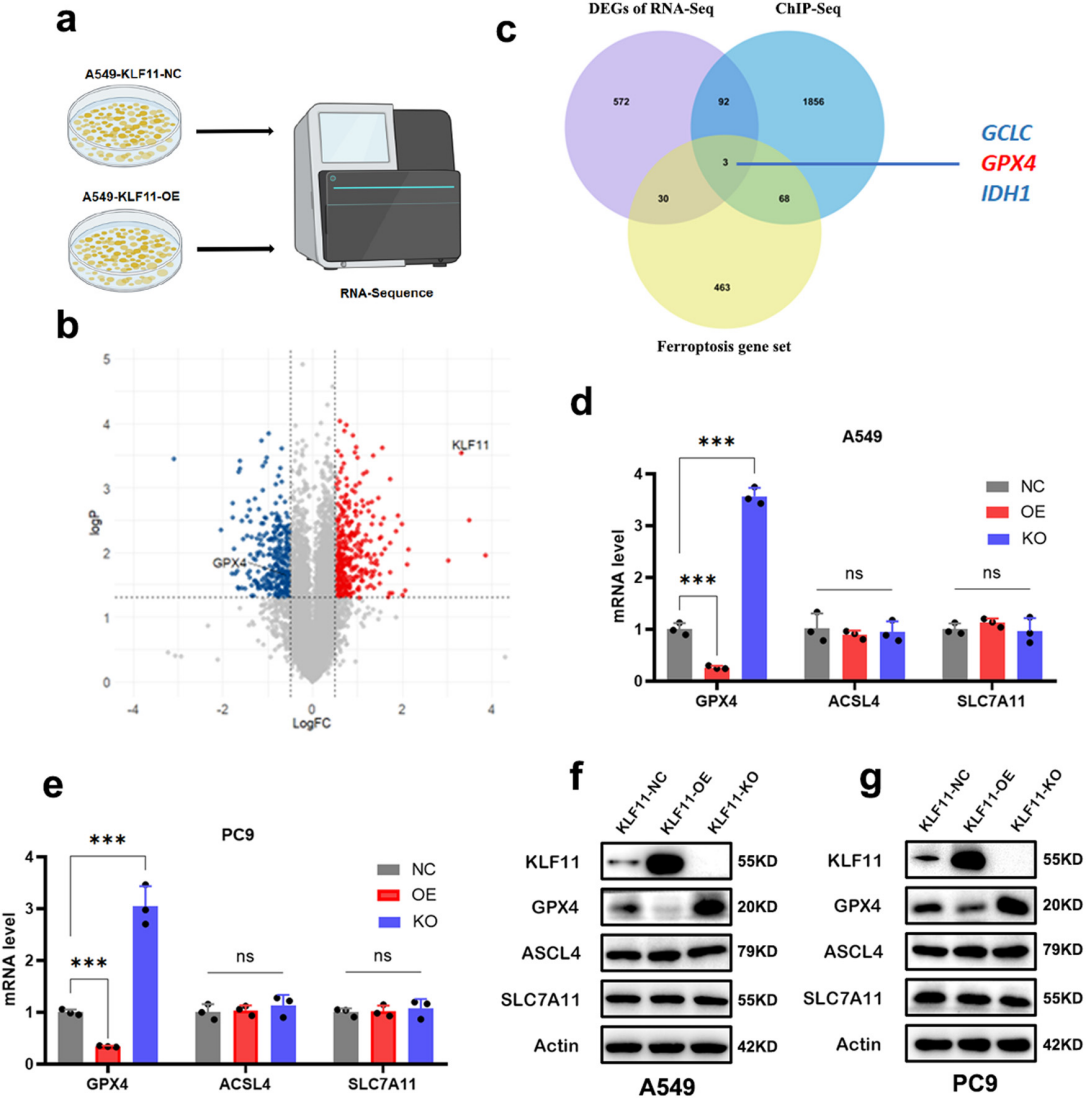
Identification of GPX4 as the target molecule of KLF11 to regulate ferroptosis. **a** Schematic diagram of RNA-seq screening workflow in A549 cell line transfected with KLF11-NC and KLF11-OE. **b** Volcano plot showing differential expression of genes between the A549-KLF11-NC and KLF11-OE. **c** Venn plot showing the intersection of predicted targets of KLF11. **d**, **e** qRT-PCR results showed differences in RNA levels of three key molecules (GPX4, SLC7A11 and ASCL4) of ferroptosis between the KLF11-NC, KLF11-OE and KLF11-KO of A549 and PC9 cell lines. **f**, **g** Western blot results showed differences in protein levels of GPX4, SLC7A11, and ASCL4 of ferroptosis between the KLF11-NC, KLF11-OE, and KLF11-KO of A549 and PC9 cell lines.

To explore the downstream signaling molecules of KLF11 promoting ferroptosis, we conducted RNA-seq in KLF11-NC and KLF11-OE cells respectively (Fig. 4a) and displayed differentially expressed genes (Fig. 4b) Then, we took the intersection of the results between RNA-Seq, ChIP-Seq, and the specific gene set of ferroptosis (Obtained from public databases, http://www.zhounan.org/ferrdb/current/) (Fig. 4c). The results indicated that GPX4 was significantly down-regulated upon KLF11 overexpression, implying that GPX4 may be a target molecule of KLF11. We further verified the effect of KLF11 on GPX4 expression by qRT-PCR and western blot and found overexpression of KLF11 decreased the GPX4 level, while KLF11 knockout led to the upregulation of GPX4 (Fig. 4d–g and Supplementary Fig. 1b, c). However, the level of other ferroptosis key regulators, including ACSL4 and SLC7A11 were not affected (Fig. 4d–g). These results suggest that KLF11 may be involved in LUAD ferroptosis by regulating the expression of GPX4.

### KLF11 negatively regulates the expression of GPX4 by binding to and repressing its promoter

**Fig. 5:**
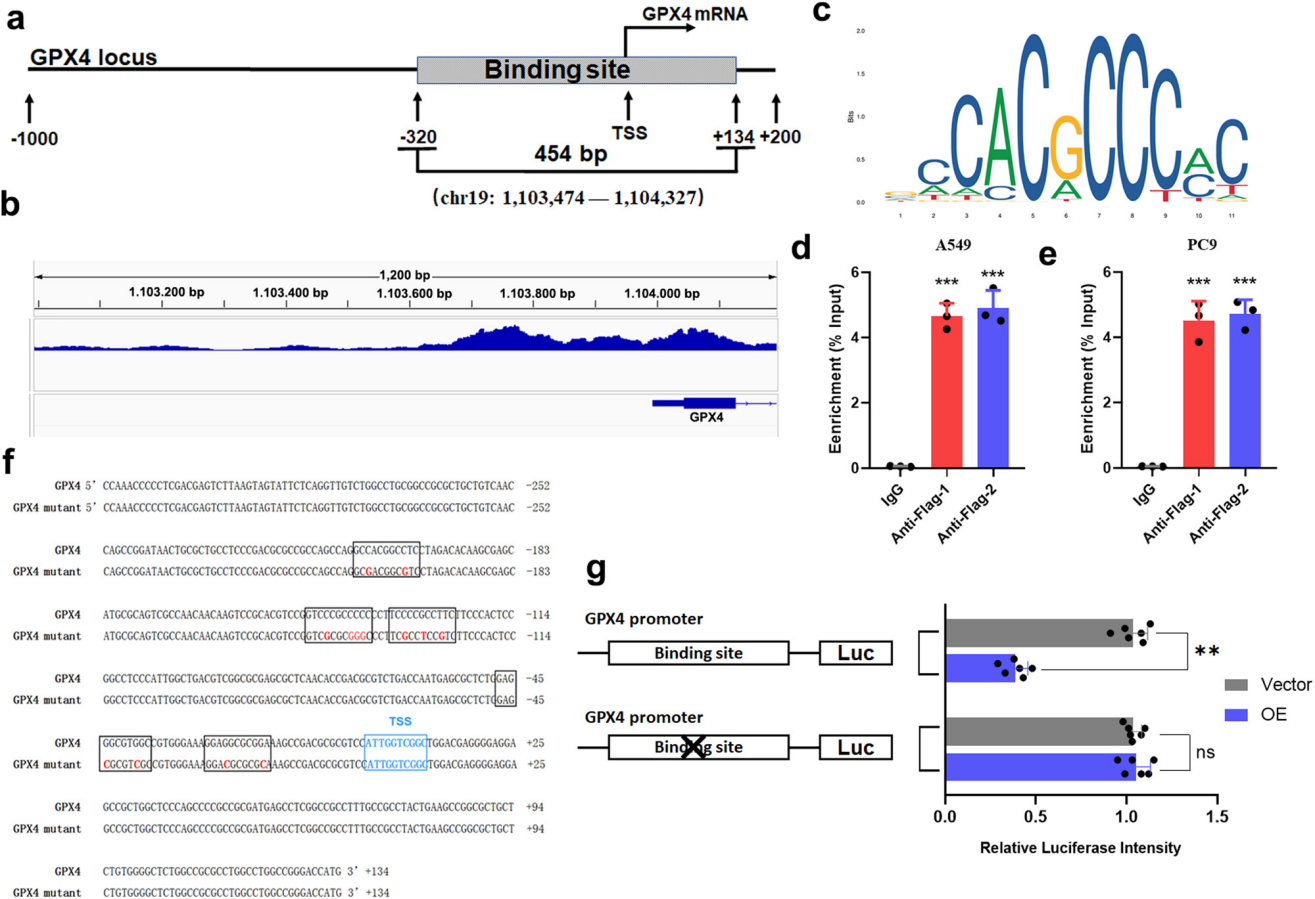
KLF11 binds to the GPX4 promoter region and represses its transcription. **a**, **b** Genome-wide data from the ENCODE project and our ChIP-seq results show the solid KLF11-binding peak in the promoter region close to the TSS of GPX4. **c** Characteristic sequences predicted by JASPAR for transcriptional binding of KLF11. **d**, **e** ChIP-qPCR assay depicting the enrichments of KLF11 binding in A549 and PC9. **f** Schematic diagram showing the possible binding sites of KLF11 to the critical region of the GPX4 promoter are mutated. **g** Dual luciferase activity assays to analyze the fluorescence intensity of KLF11-NC and KLF11-OE with or without mutations in the GPX4 promoter region.

As a transcription factor, we speculate that KLF11 transcriptionally suppresses GPX4 by binding with its promoters. To validate this hypothesis, We subsequently conducted Genome-wide ChIP-seq as well as analyzed the genome-wide data from the ENCODE project. Consistently, our results revealed a very strong KLF11-binding peak in a GPX4 promoter region proximal to the transcription start site (TSS). We identified this peak region of approximately 454 bp in length as the binding site (BS) in GPX4 (Fig. 5a–c). The ChIP-PCR assay also showed significant enrichments of KLF11 binging in both A549 and PC9 cell lines (Fig. 5d, e). These results establish GPX4 as a direct transcriptional target of KLF11.

To investigate whether KLF11 binging to the GPX4 promoter is functional, We cloned the human GPX4 promoter sequences into the PGL4-luciferase reporter vector for luciferase reporter activity experiments. As a negative control, the sites where KLF11 binds with high probability in the BS using the consensus motifs on the JASPAR were predicted and modified (Fig. 5f). As a result, KLF11 overexpression in 293T cells remarkably attenuated the activity of the luciferase reporter containing the wild-type (WT) GPX4 promoter sequences. And yet, the reporter with mutant-type (MT) GPX4 promoter constructs did not display a luciferase drop in response to KLF11 overexpression (Fig. 5g). The above outcomes revealed the mechanism of interaction between KLF11 and GPX4., suggesting that KLF11 strongly binds to the GPX4 promoter and inhibits GPX4 transcription.

### GPX4 expression antagonizes KLF11’s ability to promote ferroptosis, inhibit cell proliferation, and increase chemotherapy sensitivity

**Fig. 6:**
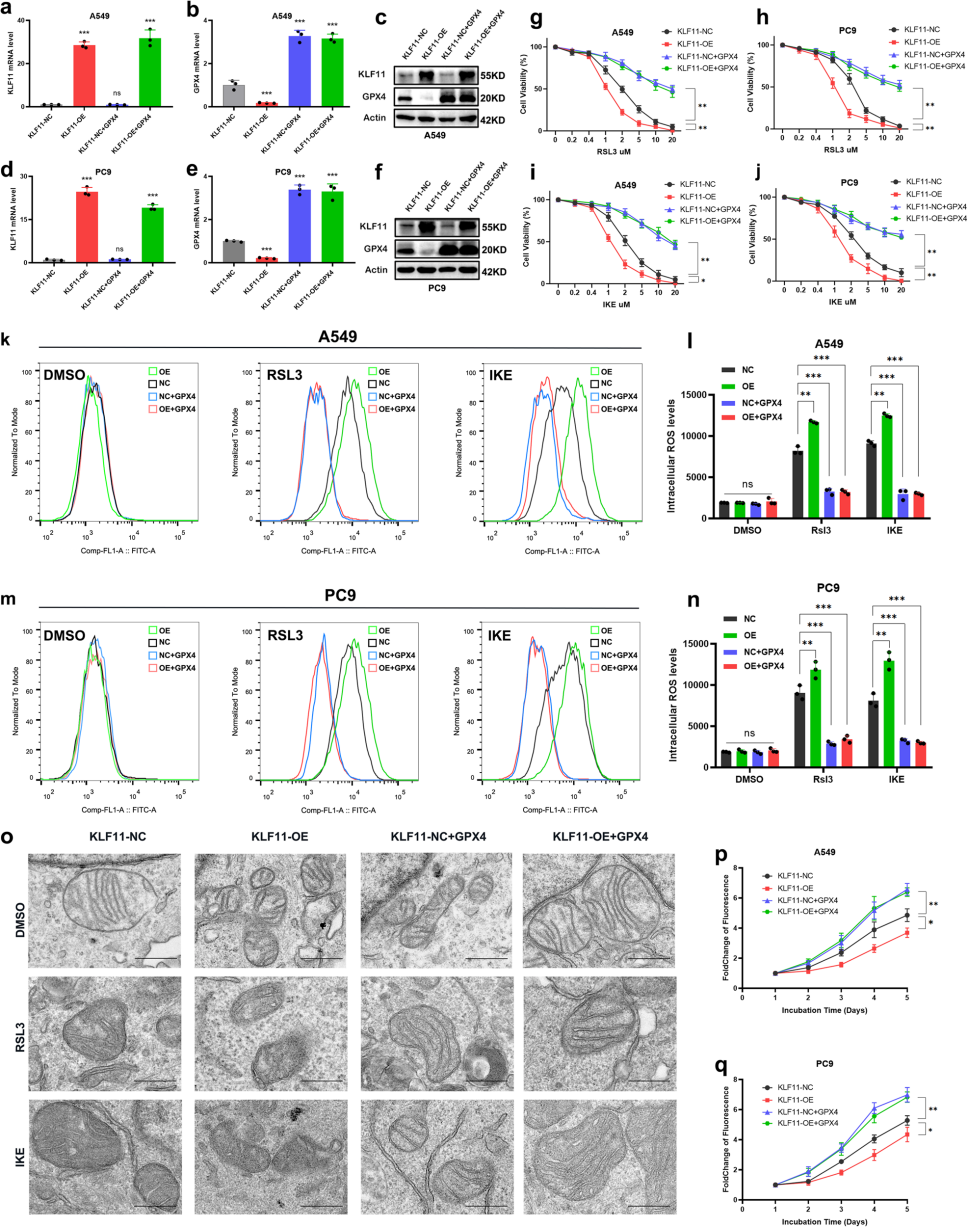
Restoring GPX4 expression antagonizes KLF11’s ability to promote ferroptosis and inhibit cell proliferation. **a**–**f** qRT-PCR and western blot confirming the restored GPX4 expression in A549 and PC9 cells transfected with KLF11-NC and KLF11-OE. **g**–**j** Dose-toxicity curves showing the viability of A549 and PC9 cells transfected with KLF11-NC, KLF11-OE, KLF11-NC+GPX4, and KLF11-OE+GPX4 upon RSL3 or IKE treatment at the indicated concentrations for 24 h. **k**–**n** Lipid peroxidation was measured by flow cytometry after C11-BODIPY staining in KLF11-NC, KLF11-OE, KLF11-NC+GPX4, and KLF11-OE+GPX4 treatment by RSL3 or IKE. **o** Characteristic alterations of mitochondria in A549 cells treated with RSL3 or IKE analyzed by TEM after restored GPX4 expression, Scale bars: 50 nm. **p**, **q** GPX4 expression promotes the proliferation of A549 and PC9 cells inhibited by KLF11.

**Fig. 7:**
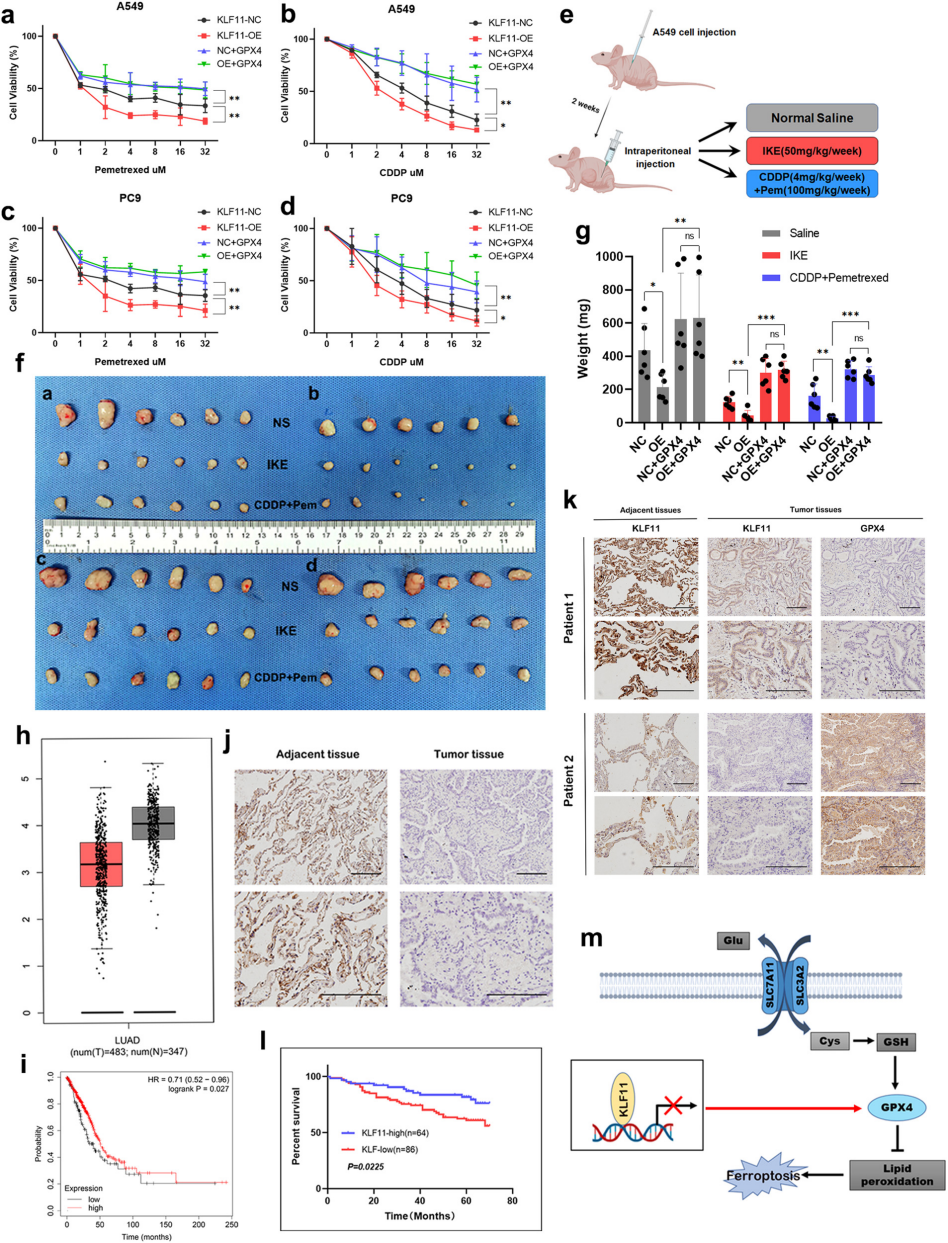
GPX4 expression antagonizes KLF11’s ability to increase chemotherapy sensitivity in vitro and in vivo, KLF11 is lowly expressed in LUAD and positively correlates with prognosis. **a**–**d** Dose-toxicity curves showing the viability of A549 and PC9 cells transfected with KLF11-NC, KLF11-OE, KLF11-NC+GPX4, and KLF11-OE+GPX4 upon CDDP or Pem treatment at the indicated concentrations for 48 h. **e** Schematic diagram showing the process of subcutaneous tumor formation in nude mice and the treatment measures. **f** Representative tumors formed in nude mice by KLF11-NC(a), KLF11-OE(b), KLF11-NC+GPX4(c), and KLF11-OE+GPX4(d) cells upon IKE or CDDP+Pem treatment. **g** The weight of the final tumor formed between different groups. **h** The expression of KLF11 between adjacent normal tissues and tumor tissues from patients with LUAD in our hospital. **i** The expression of KLF11 was negatively correlated with GPX4. **j** The low expression of KLF11 in LUAD compared to normal lung tissue was verified in the TCGA database. **k**, **l** Both our cohort and the TCGA cohort showed a positive correlation between KLF11 expression and prognosis in LUAD patients. **m** Mechanism of KLF11 regulation of GPX4 transcription.

The above results indicate that KLF11 promotes ferroptosis in tumor cells by inhibiting GPX4 transcription. Therefore, we next restored GPX4 expression in the KLF11-OE and NC cell lines (Fig. 6a–f). We found that high expression of GPX4 caused significant resistance of LUAD cells to FINs (Fig. 6g–j). In addition, high GPX4 expression also resulted in a significant reduction in intracellular ROS levels in KLF-OE and NC cell lines after FINs treatment (Fig. 6k–n). Moreover, TEM assay revealed that after FINs treatment, cells restoring GPX4 expression exhibited resistance features to ferroptosis, characterized by an almost normal mitochondrial morphology (Fig. 6o). The difference in FINs sensitivity between the two cell lines was diminished. Regarding the effect on the proliferation, GPX4 expression significantly enhanced the proliferation ability in both KLF11-OE and NC LUAD cell lines (Fig. 6p–q). Intriguingly, reversion to GPX4 expression significantly attenuated the toxic response of KLF11-promoted chemotherapy to lung adenocarcinoma cells (Fig. 7a–d). These results suggest that GPX4 can antagonize the function of KLF11-mediated promotion of ferroptosis and inhibition of cell proliferation.

### KLF11 inhibits tumor formation and can be antagonized by GPX4 in vivo

To validate the results obtained above in vivo, Subcutaneous tumor formation was conducted subcutaneously by injecting different A549 cells (KLF11-NC, KLF11-OE, KLF11-NC+GPX4, KLF11-OE+GPX4) into nude mice (Fig. 7e). Throughout the period, we regularly observed tumor growth while measuring and recording tumor size weekly. After 4 weeks, the nude mice were euthanized and the tumors removed, and all the tumor weights were measured. The results showed that tumor formation in nude mice injected with KLF11-OE cells was significantly suppressed compared to the KLF11-NC group (Fig. 7f_a-b_–g). Moreover, consistent with in vitro experiments, it is interesting to note that this inhibition is suppressed by the restoring expression of GPX4 (Fig. 7f_c-d_–g). Furthermore, different groups of nude mice were treated with FINs or chemotherapeutic agents during tumor proliferation, and it was found that the tumors in the KLF11-OE group regressed significantly compared to KLF11-NC+GPX4 and KLF11-OE+GPX4 groups (Fig. 7f, g).

### KLF11 lowly expresses in tumor tissues and negatively correlated with GPX4 expression in clinical samples

To explore the clinical significance of KLF11 in LUAD, we searched the TCGA database and found that KLF11 was lowly expressed in LUAD tissues (Fig. 7h), and its expression level was positively correlated with the prognosis of LUAD patients (Fig. 7i). To verify the results, we retrospectively analyzed the clinicopathological characteristics and prognosis of 150 LUAD patients with complete follow-up data in 2014 (Supplementary Table S4). IHC staining also showed a relatively low KLF11 expression in LUAD tissues while this expression was remarkably high in nontumorous tissues (Fig. 7j). Moreover, the level of KLF11 was negatively correlated with the expression of GPX4 (Fig. 7k, Supplementary Fig. 2a, and Supplementary Table S4). Additionally, Kaplan–Meier analyses showed that KLF11 low expression significantly correlates with poor overall survival of LUAD patients (Fig. 7l), and a multivariate Cox proportional hazards model indicated that low expression of KLF11 was an independent prognostic predictor of OS (*P* = 0.043, Supplementary Table S5). A total of 48 patients out of 150 LUAD patients experienced postoperative adjuvant chemotherapy(ACT) from the 150 LUAD patients. We defined those who relapsed before the follow-up cutoff as chemoresistant and those who did not relapse as chemo-sensitive, the clinicopathological characteristics of the 48 cases were shown in Supplementary Table S6. We found low KLF11 expression as well as high GPX4 expression was associated with chemoresistance (Supplementary Table S6), and KLF11 expression was negatively associated with GPX4 expression (Supplementary Fig. 2b).

## Discussion

Ferroptosis is an iron-dependent regulated cell death mode, identified in 2012, markered by the excessive amount of cellular lipid peroxidation, eventually leading to the ruptured plasma membrane and cell death^12,13^. Increasing evidence has demonstrated that dysregulation of ferroptosis plays a vital role in human cancer development^14–16^. In this study, we revealed that KLF11 is involved in the regulation of ferroptosis. Our results showed that ferroptosis stress leads to an increase in KLF11 expression, which can enhance the cell sensitivity to ferroptosis and chemotherapy by inhibiting GPX4 transcription (Fig. 7m).

The KLFs are a family of transcription factors that are involved in various pathophysiological processes. Previously, our group has paid attention to the KLFs family and has carried out research in the early stage. Coincidentally, we have also found the involvement of KLF11, a member of the KLFs family, in the ferroptosis of LUAD by high-throughput sequencing. Moreover, the public databases showed aberrant expression of KLF11 in multiple tumor tissues and KLF11 has been shown to play a crucial role during tumorigenesis and development^17–19^. Therefore, we conducted this study to explore the role of KLF11 in the ferroptosis and chemotherapy of LUAD. Structurally, KLF11 mediates transcriptional repression as it contains three repressive structural domains (R1, R2, and R3) at its N-terminal transcriptional regulatory region^17,20,21^. The R1 structural domain is an α-helical repressor motif (HRM) that represses transcriptional activation of target genes through interaction with the co-repressor mSin3A^21^. Many studies have also confirmed that KLF11 is a repressive transcription factor^18,19,22^. Mechanistically, KLF11 recruits mSin3a to repress the transcription of Smad7 promoter through GC-rich sites, then deregulates the negative feedback loop on the TGF-β signaling pathway and promoting the activation of the anti-proliferative signaling pathway, thereby inhibiting epithelial cell growth^20,23^. In addition, KLF11 expression can suppress the expression of SOD2 and Catalase1, which are key genes for oxidative stress elimination, inhibited cell growth and induced apoptosis in vitro and in vivo^19^. Therefore, KLF11 can affect cell growth and carcinogenesis through multiple mechanisms^17^, and whether it participates in ferroptosis has not been determined. In our study, KLF11 expression was found to be upregulated by high-throughput sequencing in LUAD cells after treatment with FINs, and altering KLF11 levels significantly affected the sensitivity of LUAD cells to FINs, establishing that KLF11 is involved in the regulation of ferroptosis in LUAD. Since KLF11 is a functional transcription factor and is engaged in ferroptosis, we assumed that KLF11 can regulate the key molecule of the ferroptosis pathway. As previous studies demonstrated, ferroptosis can be regulated by multiple critical factors^10,24^. As a key molecule in the endogenous pathway of ferroptosis, GPX4 is considered to be the antioxidant enzyme that utilizes reduced glutathione (GSH) as a cofactor to detoxify lipid peroxidation, thereby acting as the gatekeeper to inhibiting the occurrence of ferroptosis^8,10,25–27^. Cancer cells can also counter their susceptibility to ferroptosis by regulating GPX4 in the ferroptosis pathway. The genetic suppression of GPX4 was proven to induce ferroptosis in tumor cells and inhibit tumor growth^10^. However, the regulatory mechanism of GPX4 during cancer cell ferroptosis remains to be further investigated. As indicated by western blot, ChIP-Seq and dual luciferase reporter assays, our results suggest that GPX4 is a transcriptional regulatory target molecule of KLF11, which acts as a repressive transcription factor by binding to the promoter region of GPX4, thereby inhibiting its transcription. Moreover, GPX4 expression can antagonize KLF11’s ability to promote ferroptosis, suggesting that GPX4 is a specific target of KLF11 in the ferroptosis pathway. Additionally, considering that the luciferase levels of WT and MT GPX4 promoters were affected by FINs treatment, we have conducted experiments to compare the luciferase level of WT and MT GPX4 promoters with non-treating and FINs-treating. We found that in the GPX4 WT group, the luciferase levels of cells in the FINs-treated group were slightly elevated compared with the DMSO group, but not statistically significant. Similarly, the fluorescence values of cells in the MT group were mildly elevated after FINs treatment, and the increase was more significant than in the WT group, but the results were also not statistically significant (Supplementary Fig. 1e), suggesting that the regulatory mechanism of GPX4 is complex, and there may be other mechanisms besides the regulation of KLF11. Considering intracellular iron ions are involved in another ferroptosis regulatory mechanism, there is no evidence that modulation of GPX4 can affect intracellular iron ions’ levels. We examined the iron ion concentration in KLF11 overexpression and knock-out A549 and PC-9 cells and found that the different KLF11 expressions did not affect the intracellular iron ion levels (Supplementary Fig. 1d).

Recently, ferroptosis therapy showed synergistic affection in cancer treatment with traditional chemoradiotherapy and immunotherapy^16^. At present, chemotherapy remains the main treatment for patients with LUAD. Unfortunately, the development of tumor chemotherapy resistance is one of the important reasons for the failure of antitumor therapy. Since ferroptosis can be induced by chemotherapy, its regulation is closely related to tumor progression and chemotherapy resistance^7,9^. Recent studies have indicated that GPX4 plays an essential role in tumor resistance to chemotherapy or radiotherapy^9,28^. Since our study has confirmed that KLF11 promotes LUAD ferroptosis by suppressing GPX4, and It has been shown that KLF11 is associated with inferior chemotherapy response in sarcomas^18^. We speculate that KLF11 may also be involved in the resistance of LUAD to chemotherapy through the regulation of GPX4 expression. Consistent with the assumption, our results showed that KLF11 expression could inhibit the proliferation of LUAD cells while enhancing the toxic response of LUAD to chemotherapeutic drugs in vitro and in vivo. More importantly, the restored expression of GPX4 was able to suppress the enhanced chemosensitivity of LUAD by KLF11, indicating that KLF11 can alleviate the resistance of cells to chemotherapeutic drugs by enhancing their ferroptosis. Clinicopathologically, we also found that KLF11 was low expressed in the LUAD tumor tissues compared to adjacent normal tissues, and the level of KLF11 was negatively correlated with the expression of GPX4 but positively correlated with the prognosis of LUAD patients. Indeed, tissue microarray is more suitable in immunohistochemical experiments to detect the protein expression in a lot of tissue samples.

Taken together, our data establish KLF11 as a ferroptosis regulator with therapeutic potential and clinical value as a prognostic factor.

## Materials and methods

### Cell lines

The human LUAD cell lines A549, PC9 and human embryonic kidney 293 (HEK293T) cells were cultured in high-glucose Dulbecco’s modified Eagle’s medium (DMEM; Hyclone, Logan, UT, USA) supplemented with 10% fetal bovine serum (EveryGreen, Zhejiang, China) and 100 U/mL penicillin/streptomycin/amphotericin B (Sangon Biotech, Shanghai, China). All cells were purchased from the Chinese Academy of Science Cell Bank and were cultured in a 37 °C incubator in a humidified 5% CO_2_ atmosphere.

### Patients and tumor specimens

Archival specimens were obtained from 150 LUAD patients in 2014 with informed consent at the Department of Thoracic Surgery, Zhongshan Hospital, Fudan University. All specimens from these patients underwent available formalin-fixed, paraffin-embedded, and had complete clinicopathology and follow-up data. The tumor stage was determined according to the tumor/lymph node/metastasis (TNM) classification using the eighth edition of the International Union Against Cancer (UICC) *Cancer Staging Manual*, and the pathologic classification was based on World Health Organization (WHO) criteria. Follow-up was completed in November 2019. Overall survival (OS) was defined as the interval between surgery and death or the last observation for surviving patients. This study was approved by Zhongshan Hospital Research Ethics Committee, Fudan University (B2022-180R).

### Compounds

RSL3 (T3646), IKE (T1765), ferrostatin-1 (T6500), deferoxamine (DFO; T1637), Cis‐Diamminedichloride platinum (CDDP, T1564), and Pemetrexed (Pem, T6226) were provided by Topscience (Shanghai, China). The first four compounds were dissolved in DMSO (Beyotime, Shanghai, China) and the last two in PBS (Beyotime), according to their solubility, and then stored at −80 °C.

### Cell viability assays

For cytotoxicity assays, 3000 cells per well were seeded in quintuplicate in 96-well plates and incubated for 24 h. Cells were treated with different doses of FINs or chemotherapy drugs for 48 h as required. For proliferation assays, the cells were seeded at a density of 1000 cells per well and incubated for 0, 24, 48, 72, 96, and 120 h at 37 °C. Cell viability was measured with CCK-8 (Beyotime) according to the manufacturer’s instructions.

### Chromatin immunoprecipitation (ChIP) assays

ChIP assay is performed using the SimpleChIP® Plus Enzymatic Chromatin IP Kit (Magnetic Beads) (CST). Firstly, cells were crossed-linked, membrane lysed, and treated with micrococcal nuclease to fragment chromatin. Then digested and cross-linked chromatin was incubated with Flag-Tag Antibody (CST). Histone H3 antibody (CST) and human IgG (CST) were used as the positive and negative control respectively. Antibodies are listed in detail in Supplementary Table S2. DNA was eluted from the beads after thoroughly washing and used in qRT-PCR.

### Lentiviral infections

For the establishment of cell lines (A549 and PC9) with stable KLF11 or GPX4 overexpression, the lentivirus vectors KLF11 and GPX4, and corresponding negative control sequences, were obtained from Genechem Technology. In addition, the viral vector for knockout of KLF11 expression and the negative control sequences were also obtained from Genechem Technology (Supplementary Table S3). The summary procedure is as follows, a total of 5 × 10^4^ cells (A549 and PC9) were seeded into 12-well plates. Twenty-four hours later, lentivirus was added at an MOI of 10, cells were cultured in the complete medium containing 5 mg/mL polybrene for 12 h, and the culture medium was replaced with fresh medium. At 72 h after transduction, the cells were subsequently propagated in the selection medium containing 2.5 mM puromycin (Hanyin Technology).

### Lipid peroxidation detection

Cells were seeded in 12-well plates and treated with drugs for an appropriate time on the next day, and then were incubated with 1 ml of fresh medium containing 5 μM of BODIPY 581/591 C11 (Invitrogen) for 30 min. Cells were then trypsinized, washed, and resuspended in 0.5 ml of PBS for flow cytometry analysis. A minimum of 20,000 cells were analyzed per condition. FlowJo software was used to analyze the results (TreeStar, Woodburn, OR, USA).

### Intracellular iron concentration determination

The iron ion concentration was detected using the manufacturer of Iron Assay Kit (ab83366, Abcam), and the procedure of the assay was based on the protocol according to the Kit.

### Quantitative real-time PCR

RNA extraction and quantitative real-time PCR (qRT-PCR) were performed as previously described^29^. Briefly, Total RNA was extracted from tissues or cells with TRIzol reagent (TIANGEN), and cDNA was synthesized with a Hifair II First-Strand cDNA Synthesis Kit (gDNA Digester Plus, YEASEN, China). qRT-PCR was performed with a Hifair III One-Step run on an ABI QuantStudio 5 real-time PCR system (Thermo Fisher, USA). The threshold cycle (Ct) values for each gene were normalized to those of Actin as an endogenous calibrator, and the 2^(−△△CT) method was used for quantitative analysis. The primers of each gene were synthesized by Sangon Biotech and are listed in Supplementary Table S1.

### Western blotting

Briefly, cells were lysed in RIPA buffer (Beyotime) containing protease and phosphatase inhibitor cocktail (Beyotime). The obtained proteins were quantified by quantified with an Enhanced BCA Protein Assay Kit (Beyotime) and were then boiled in 5×SDS-PAGE loading buffer (EpiZyme, Shanghai, China) for 10 min at 100 °C. Next, proteins were subjected to SDS-PAGE for western blotting. The following antibodies were used: KLF11 (1:1000, #AF0315), GPX4 (1:1000, #DF6701), and SLC7A11 (1:1000, #DF12509) from Affinity Biosciences, ASCL4 (1:1000, #abs106075) from Absin, and β-actin (1:10000, # D191047) from Sangon. The uncropped scans for the bands were shown in Supplementary Fig. 3.

### Immunohistochemistry (IHC)

KLF11 antibody (1:100, Affinity, #AF0315), GPX4 antibody (1:100, Affinity, #DF6701), and GTVisionTM III Detection System (Gene Tech, Shanghai, China) were used for immunohistochemical staining. The intensity of positive staining was measured as described^30^ and their expression was classified into high and low groups according to a cutoff value of 40%.

### Dual-luciferase reporter assays

We cloned the sequence (−1000 ~ 200 bp around the Transcription Starting Site), containing 5’-UTR, the first exon, and part of the first intron of GPX4 promoter region, into the phy-811@7 dual luciferase reporter vector (Hanyin Technology, Shanghai, China). HEK293T cells were seeded on a polylysine-treated 24-well plate at 60%–70% confluence. After 24 h, the cells were co-transfected with 200 nM KLF11 mimics and 400 ng of the wild-type or mutant plasmids constructed as above with Lipo8000 (Beyotime) as the transfection reagent. Forty-eight hours later, the cells were collected, and the dual-luciferase reporter assays were conducted with a Luciferase Reporter Gene Assay Kit (Beyotime). Lastly, luciferase activity was detected with a Microplate spectrophotometer (Bio-Rad, Hercules, CA, USA).

### Transmission electron microscopy

Cells cultured in 6-cm dishes were fixed with a solution containing 2.5% glutaraldehyde. After being washed in 0.1 M phosphate buffer solution (PBS, pH 7.4) three times, cells were postfixed with phosphate buffer containing 1% osmic acid, followed by washing in 0.1 M PBS (pH 7.4) another three times. Cells were dehydrated and embedded, and then incubated in a 60 °C oven for 48 h. Ultrathin sections were prepared and stained with lead citrate and uranyl acetate. After drying overnight, the sections were examined with a Hitachi transmission electron microscope(Hitachi, Japan).

### Tumor xenograft model in Nude mice

The animal experiments were conducted in compliance with the policies of the Animal Ethics Committee of Zhongshan Hospital. Six million A549 cells with different treatments were resuspended in 300 μl culture media and injected subcutaneously into the left flank of 4w-old male BALB/c nude mice. The xenograft tumors were gathered 4 weeks after implantation. Tumor weights were measured, and tumor volumes were determined as (length × width^2^)/2.

### Statistics and Reproducibility

All statistical analyses were conducted in GraphPad Prism software (8.0) and R software. Unpaired Student’s t-tests and Wilcoxon test were performed to compare continuous variables between two groups. Correlation analysis between KLF11 and GPX4 was performed using Spearman coefficient tests. Survival analysis was calculated using the Kaplan–Meier method and the log-rank test. A Cox proportional hazards regression model was used to analyze independent prognostic factors. The results are presented as means and the error bars represent the standard deviation unless stated otherwise. The *p* values were all two-tailed, and *p* < 0.05 was considered significant: *p  <  0.05, **p  <  0.01, ***p  <  0.001, ns, not significant. All experiments in this study were repeated three times.

### Reporting summary

Further information on research design is available in the Nature Portfolio Reporting Summary linked to this article.

## Supplementary information

Supplementary Information

Reporting Summary

## Data Availability

RNA-Seq data and numerical source data have been deposited in Figshare (10.6084/m9.figshare.21026443, 10.6084/m9.figshare.22767161, and 10.6084/m9.figshare.22820801). Other source data supporting the findings of this study are available from the corresponding author upon reasonable request.

## References

[1] Siegel RL, Miller KD, Fuchs HE, Jemal A (2022). Cancer statistics, 2022. CA Cancer J. Clin..

[2] Travis WD (2015). The 2015 World Health Organization classification of lung tumors: impact of genetic, clinical and radiologic advances since the 2004 classification. J. Thorac. Oncol..

[3] Boumahdi S, de Sauvage FJ (2020). The great escape: tumour cell plasticity in resistance to targeted therapy. Nat. Rev. Drug Discov..

[4] Arriagada R (2004). Cisplatin-based adjuvant chemotherapy in patients with completely resected non-small-cell lung cancer. N. Engl. J. Med.

[5] Herbst RS, Morgensztern D, Boshoff C (2018). The biology and management of non-small cell lung cancer. Nature.

[6] Stockwell BR (2022). Ferroptosis turns 10: emerging mechanisms, physiological functions, and therapeutic applications. Cell.

[7] Zhang C, Liu X, Jin S, Chen Y, Guo R (2022). Ferroptosis in cancer therapy: a novel approach to reversing drug resistance. Mol. Cancer.

[8] Zhang Y (2021). mTORC1 couples cyst(e)ine availability with GPX4 protein synthesis and ferroptosis regulation. Nat. Commun..

[9] Wang, Y. et al. Wnt/beta-catenin signaling confers ferroptosis resistance by targeting GPX4 in gastric cancer. *Cell Death Differ.*, 10.1038/s41418-022-01008-w (2022). 10.1038/s41418-022-01008-w PMC9613693 35534546

[10] Yang WS (2014). Regulation of ferroptotic cancer cell death by GPX4. Cell.

[11] Xie Y (2016). Ferroptosis: process and function. Cell Death Differ..

[12] Dixon SJ (2012). Ferroptosis: an iron-dependent form of nonapoptotic cell death. Cell.

[13] Gao W, Wang X, Zhou Y, Wang X, Yu Y (2022). Autophagy, ferroptosis, pyroptosis, and necroptosis in tumor immunotherapy. Signal Transduct. Target Ther..

[14] Jiang L (2015). Ferroptosis as a p53-mediated activity during tumour suppression. Nature.

[15] Zhang Y (2018). BAP1 links metabolic regulation of ferroptosis to tumour suppression. Nat. Cell Biol..

[16] Chen X, Kang R, Kroemer G, Tang D (2021). Broadening horizons: the role of ferroptosis in cancer. Nat. Rev. Clin. Oncol..

[17] Lin, L., Mahner, S., Jeschke, U. & Hester, A. The distinct roles of transcriptional factor KLF11 in normal cell growth regulation and cancer as a mediator of TGF-β signaling pathway. *Int. J. Mol. Sci.* **21**, 10.3390/ijms21082928 (2020). 10.3390/ijms21082928 PMC7215894 32331236

[18] Wang, Y. et al. Genome-wide CRISPR-Cas9 screen identified KLF11 as a druggable suppressor for sarcoma cancer stem cells. *Sci. Adv.* **7**, 10.1126/sciadv.abe3445 (2021). 10.1126/sciadv.abe3445 PMC7840125 33571129

[19] Fernandez-Zapico ME (2003). An mSin3A interaction domain links the transcriptional activity of KLF11 with its role in growth regulation. Embo J..

[20] Cook T, Gebelein B, Mesa K, Mladek A, Urrutia R (1998). Molecular cloning and characterization of TIEG2 reveals a new subfamily of transforming growth factor-beta-inducible Sp1-like zinc finger-encoding genes involved in the regulation of cell growth. J. Biol. Chem..

[21] Zhang JS (2001). A conserved alpha-helical motif mediates the interaction of Sp1-like transcriptional repressors with the corepressor mSin3A. Mol. Cell Biol..

[22] Zhao, G. et al. KLF11 protects against abdominal aortic aneurysm through inhibition of endothelial cell dysfunction. *JCI Insight* **6**, 10.1172/jci.insight.141673 (2021). 10.1172/jci.insight.141673 PMC8021107 33507881

[23] Ellenrieder V (2004). KLF11 mediates a critical mechanism in TGF-beta signaling that is inactivated by Erk-MAPK in pancreatic cancer cells. Gastroenterology.

[24] Koppula P, Zhuang L, Gan B (2021). Cystine transporter SLC7A11/xCT in cancer: ferroptosis, nutrient dependency, and cancer therapy. Protein Cell.

[25] Li J (2020). Ferroptosis: past, present and future. Cell Death Dis..

[26] Li D, Li Y (2020). The interaction between ferroptosis and lipid metabolism in cancer. Signal Transduct. Target Ther..

[27] Forcina GC, Dixon SJ (2019). GPX4 at the crossroads of lipid homeostasis and ferroptosis. Proteomics.

[28] Hangauer MJ (2017). Drug-tolerant persister cancer cells are vulnerable to GPX4 inhibition. Nature.

[29] Bi G (2022). miR-6077 promotes cisplatin/pemetrexed resistance in lung adenocarcinoma via CDKN1A/cell cycle arrest and KEAP1/ferroptosis pathways. Mol. Ther. Nucleic Acids.

[30] Zhao GY, Ding JY, Lu CL, Lin ZW, Guo J (2014). The overexpression of 14-3-3ζ and Hsp27 promotes non–small cell lung cancer progression. Cancer.

