# Supplementary material for: KLF11 regulates lung adenocarcinoma ferroptosis and chemosensitivity by suppressing GPX4
Source: Commun Biol. 2023 May 29;6:570. doi: 10.1038/s42003-023-04959-z (PMC10227016; doi:10.1038/s42003-023-04959-z)
Supplement: Supplementary file 1 — Supplementary Information [file 42003_2023_4959_MOESM1_ESM.pdf]

Supplementary Figure 1

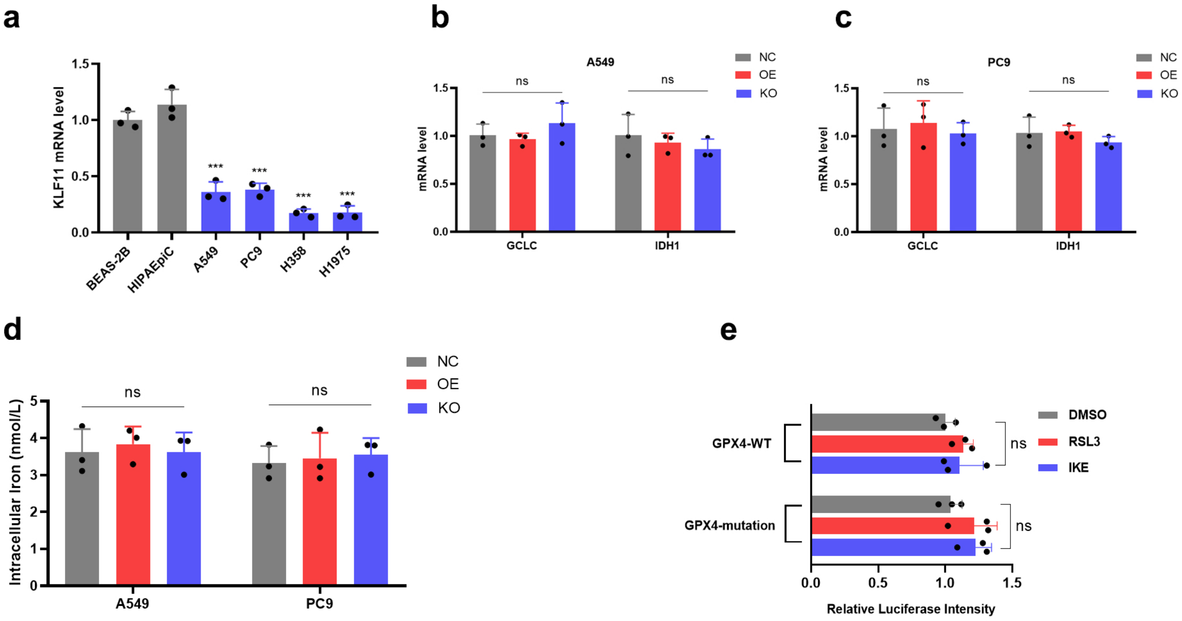

Supplementary Figure 2

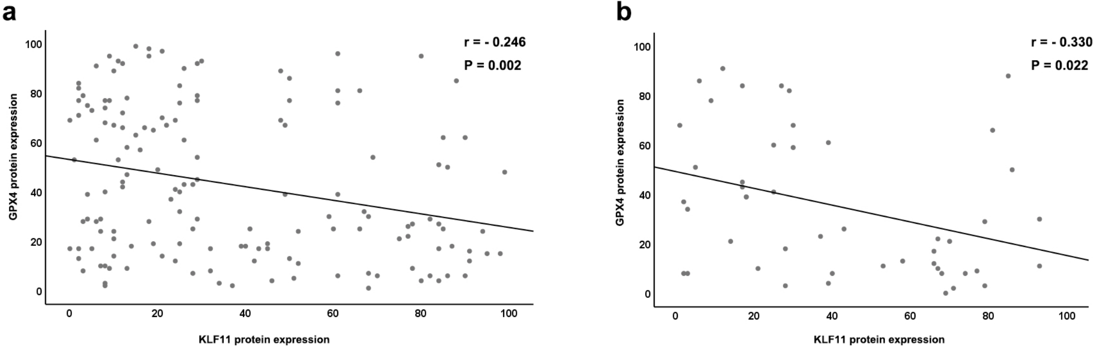

Supplementary figure 3

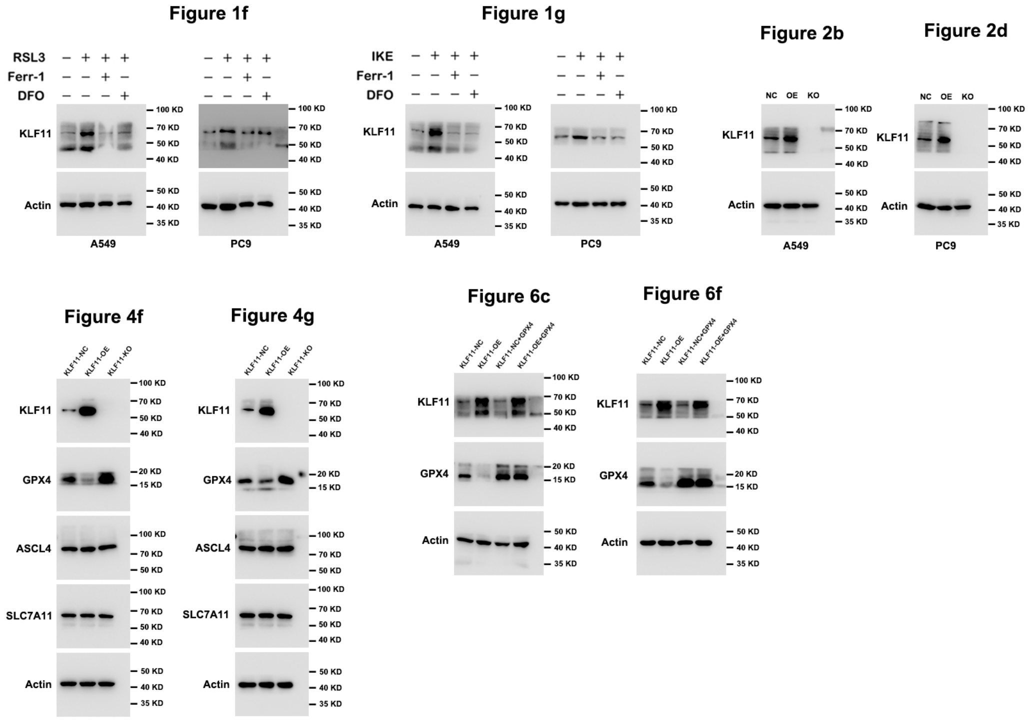

Supplementary Figure 1. (a) The expression level of KLF11 in normal lung epithelial cell lines and cancer cell lines. (b and c) The mRNA level of GCLC and IDH1 at different KLF11 levels (d) Intracellular iron ion levels in A549 and PC9 cells with different KLF11 expression. (e) The luciferase level between WT (wild type) and MT (mutation type) GPX4 promoter with non-treating and FINs-treating group.

Supplementary Figure 2. (a) Correlation of KLF11 and GPX4 expression in 150 patients with LUAD. (b) Correlation of KLF11 and GPX4 expression in 48 LUAD patients undergoing postoperative adjuvant chemotherapy (ACT).

Supplementary Figure 3. Uncropped scans for the bands shown in the manuscript.

Table S1. Sequences of Primers for Quantitative real-time-Polymerase Chain Reaction Analysis

Gene	Forward Primer: 5'-3'	Reverse Primer: 5'-3'
KLF11	GCATGACAGCGAAAGGTCTAC	GGGGTCTTATCCGCAACAGG
GPX4	AGAGATCAAAGAGTTCGCCGC	TCTTCATCCACTTCCACAGCG
GPX4(ChIP-qPCR)	CCTCCTAGACACAAGCGAGCAT	CGGCTTCAGTAGGCGGCAAA
SLC7A11	GGTGGAACGAGGAGGTGGAGAA	TTGGAGATGGTGGACACAACAGG
ASCL4	ATGTCTGCTTCTGCTGCCCAAT	AACCGCCTTCTTGCCAGTCTT
GCLC	GGCACAAGGACGTTCTCAAGT	CAGACAGGACCAACCGGAC
IDH1	AGAAGCATAATGTTGGCGTCA	CGTATGGTGCCATTTGGTGATT

Table S2. Details of antibodies used in this research

Protein	Supplier	Cat No.	Source	Application	Concentration
ACSL4	Absin	abs106075	Rabbit	western blot	1:1000
KLF11	Affinity	AF0315	Rabbit	western blot, IHC	1:1000, 1:50
Flag-Tag	CST	14793S	Rabbit	ChIP	1:50
GPX4	Affinity	DF6701	Rabbit	western blot, IHC	1:1000, 1:100
SLC7A11	Affinity	DF12509	Rabbit	western blot	1:1000
β-Actin	Sangon	D191047	Mouse	western blot	1:5000

Table S3. Sequences of sgRNA

Name	Sequence (5'-3')
sgRNA-KLF11-KO	GGAAGCGGCATGACAGCGAA

Table S4. Clinicopathologic Variables in 150 Patients With LUAD

**Variable**	**No. of Patients**	**KLF11 expression**		***P***
		**Low**	**High**	
Age				
≤62	80	42	38	0.201
> 62	70	44	26	
Gender				
Male	67	39	28	0.846
Female	83	47	36	
Smoking status				
Yes	29	20	9	0.158
No	121	66	55	
Stage				
I-II	120	62	58	0.005
III-IV	30	24	6	
Lymph node metastasis				
Yes	42	32	10	0.004
No	108	54	54	
Tumor size				
≤3cm	66	31	35	0.023
> 3cm	84	55	29	
GPX4 expression				
Low	72	27	45	<0.001
High	78	59	19	

Table S5 Univariate and Multivariate Analyses of Factors Associated With Overall Survival

Variables	Univariate analyse HR (95% CI)	*P*	Multivariate analyse HR (95% CI)	*P*
Age （> 62 vs ≤ 62）	1.261(0.706-2.252)	0.434		
Gender (Male vs Female)	2.080(1.155-3.746)	0.015	2.401(1.259-4.578)	0.008
Smoking (Smoker vs Non-smoker)	1.888(0.973-3.663)	0.060		
Tumor size (>=3cm vs <3cm)	5.298(2.368-11.854)	<0.001	3.165(1.304-7.687)	0.011
Lymph node- metastasis (Yes vs No)	6.527(3.536-12.045)	<0.001	5.645(2.475-12.880)	<0.001
Tumor stage (III-IV *versus* I-II)	3.763(2.092-6.771)	<0.001	0.492(0.215-1.129)	0.094
KLF11 expression (High vs Low)	0.482(0.254-0.916)	0.026	0.496(0.263-0.904)	0.043

Abbreviations: CI, confidence interval; HR, hazard ratio; OS, overall survival.

A Cox proportional hazards regression model was used for multivariate analysis.

Table S6. Clinicopathological characteristics of 48 LUAD patients undergoing postoperative adjuvant chemotherapy(ACT).

**Variable**	**No. of Patients**	**ACT**		***P***
		**Sensitive**	**Resistant**	
Age				
≤62	22	10	12	0.827
> 62	26	11	15	
Gender				
Male	23	8	15	0.230
Female	25	13	12	
Smoking status				
Yes	9	3	6	0.712
No	39	18	21	
Stage				
I-II	37	21	16	0.001
III-IV	11	0	11	
Lymph node metastasis				
Yes	15	1	14	< 0.001
No	33	20	13	
Tumor size				
≤3cm	21	11	10	0.029
> 3cm	27	5	22	
KLF11 expression				
Low	21	7	14	0.011
High	27	19	8	
GPX4 expression				
Low	25	16	9	0.004
High	23	5	18	
