# Supplementary material for: KLF11 regulates lung adenocarcinoma ferroptosis and chemosensitivity by suppressing GPX4
Source: Commun Biol. 2023 May 29;6:570. doi: 10.1038/s42003-023-04959-z (PMC10227016; doi:10.1038/s42003-023-04959-z)
Supplement: Supplementary file 2 — Reporting Summary [file 42003_2023_4959_MOESM2_ESM.pdf]

nature portfolio

Corresponding author(s): Cheng Zhan; Di Ge

Last updated by author(s): 2023.03.30

Reporting Summary

Nature Portfolio wishes to improve the reproducibility of the work that we publish. This form provides structure for consistency and transparency in reporting. For further information on Nature Portfolio policies, see our Editorial Policies and the Editorial Policy Checklist.

Statistics

For all statistical analyses, confirm that the following items are present in the figure legend, table legend, main text, or Methods section.

n/a	Confirmed	
☐	☒	The exact sample size (*n*) for each experimental group/condition, given as a discrete number and unit of measurement
☐	☒	A statement on whether measurements were taken from distinct samples or whether the same sample was measured repeatedly
☐	☒	The statistical test(s) used AND whether they are one- or two-sided<br>*Only common tests should be described solely by name; describe more complex techniques in the Methods section.*
☒	☐	A description of all covariates tested
☒	☐	A description of any assumptions or corrections, such as tests of normality and adjustment for multiple comparisons
☐	☒	A full description of the statistical parameters including central tendency (e.g. means) or other basic estimates (e.g. regression coefficient) AND variation (e.g. standard deviation) or associated estimates of uncertainty (e.g. confidence intervals)
☒	☐	For null hypothesis testing, the test statistic (e.g. *F*, *t*, *r*) with confidence intervals, effect sizes, degrees of freedom and *P* value noted<br>*Give P values as exact values whenever suitable.*
☒	☐	For Bayesian analysis, information on the choice of priors and Markov chain Monte Carlo settings
☒	☐	For hierarchical and complex designs, identification of the appropriate level for tests and full reporting of outcomes
☒	☐	Estimates of effect sizes (e.g. Cohen's *d*, Pearson's *r*), indicating how they were calculated

Our web collection on statistics for biologists contains articles on many of the points above.

Software and code

Policy information about availability of computer code

Data collection	BD Accuri C6 was used to collect flow cytometry data.
Data analysis	GraphPad Prism software (8.0) was used for bar/line graphs output. Microsoft Excel was used for statistical analysis. FlowJo_V10 was used for flow data analysis. The RNA-Seq data were analyzed and visualized by R software (V4.0.5) with limma, ggplot2, and seruat packages. The softwares and algorithms for data analyses in this study are all well-established from previous work. There is no unreported algorithm used in this paper. The source code for data processing are available from the corresponding author on reasonable request.

For manuscripts utilizing custom algorithms or software that are central to the research but not yet described in published literature, software must be made available to editors and reviewers. We strongly encourage code deposition in a community repository (e.g. GitHub). See the Nature Portfolio guidelines for submitting code & software for further information.

Data

Policy information about availability of data

All manuscripts must include a data availability statement. This statement should provide the following information, where applicable:

- Accession codes, unique identifiers, or web links for publicly available datasets
- A description of any restrictions on data availability
- For clinical datasets or third party data, please ensure that the statement adheres to our policy

The source data supporting the findings of this study are available from the corresponding author on reasonable request.

Human research participants

Policy information about studies involving human research participants and Sex and Gender in Research.

Reporting on sex and gender	Gender was used in this study
Population characteristics	All patients in the study were Chinese
Recruitment	We retrospectively studied 150 patients with postoperative diagnosis of lung adenocarcinoma in 2014
Ethics oversight	This study was approved by Zhongshan Hospital Research Ethics Committee, Fudan University (B2022-180R).

Note that full information on the approval of the study protocol must also be provided in the manuscript.

Field-specific reporting

Please select the one below that is the best fit for your research. If you are not sure, read the appropriate sections before making your selection.

[x] Life sciences [ ] Behavioural & social sciences [ ] Ecological, evolutionary & environmental sciences

For a reference copy of the document with all sections, see nature.com/documents/nr-reporting-summary-flat.pdf

Life sciences study design

All studies must disclose on these points even when the disclosure is negative.

Sample size	No sample size calculations were performed. Sample size was determined according to our experience as well as literature reporting in terms of specific experiment.
Data exclusions	No data was excluded from the analyses in other parts of this study.
Replication	Multiple independent repeats were included for related experiments. Major findings were performed for at least twice to make sure similar results are reproducible.
Randomization	Four-week-old male BALB/c nude mice were used for tumour xenograft experiment and assigned randomly into different treatment groups.
Blinding	For cell-based experiments, cell types were known when prepare the samples or start to treat cells at the beginning of experiments. Data measurement for cell viability, RNA-Seq, FACS or photo capture were blinded to different person who processed assay at the time.

Reporting for specific materials, systems and methods

We require information from authors about some types of materials, experimental systems and methods used in many studies. Here, indicate whether each material, system or method listed is relevant to your study. If you are not sure if a list item applies to your research, read the appropriate section before selecting a response.

Materials & experimental systems

n/a	Involved in the study	
[ ]	[x]	Antibodies
[ ]	[x]	Eukaryotic cell lines
[x]	[ ]	Palaeontology and archaeology
[ ]	[x]	Animals and other organisms
[x]	[ ]	Clinical data
[x]	[ ]	Dual use research of concern

Methods

n/a	Involved in the study	
[x]	[ ]	ChIP-seq
[ ]	[x]	Flow cytometry
[x]	[ ]	MRI-based neuroimaging

Antibodies

Antibodies used

ACSL4 Absin abs106075 Rabbit western blot 1:1000
KLF11 Affinity AF0315 Rabbit western blot, IHC 1:1000, 1:50
Flag-Tag CST 14793S Rabbit ChIP 1:50
GPX4 Affinity DF6701 Rabbit western blot, IHC 1:1000, 1:100
SLC7A11 Affinity DF12509 Rabbit western blot 1:1000

	β-Actin Sangon D191047 Mouse western blot 1:5000
Validation	ACSL4 (abs106075 , Absin), https://www.absin.cn/rabbit-acsl4-facl4-polyclonal-antibody/abs106075.html<br>KLF11 (Affinity ,AF0315), https://www.affbiotech.com/goods-220-AF0315-KLF11_Antibody.html<br>Flag-Tag (CST 14793S),https://www.cellsignal.cn/products/primary-antibodies/dykddddk-tag-d6w5b-rabbit-mab-binds-to-same-epitope-as-sigma-s-anti-flag-m2-antibody/14793?site-search-type=Products&N=4294956287&Ntt=14793s&fromPage=plp&_requestid=91223<br>GPX4 (Affinity , DF6701), https://www.affbiotech.com/goods-5503-DF6701-GPX4_Antibody.html<br>SLC7A11 (DF12509, Affinity), http://www.affbiotech.com/goods-15599-DF12509-xCT_Antibody.html;

Eukaryotic cell lines

Policy information about cell lines and Sex and Gender in Research

Cell line source(s)	The human LUAD cell lines A549 and PC9, and human embryonic kidney cell line HEK-293T were purchased from the Chinese Academy of Science Cell Bank.
Authentication	Cell lines were not authenticated.
Mycoplasma contamination	All cell lines tested negative for mycoplasma contamination.
Commonly misidentified lines<br>(See ICLAC register)	HEK-293T cells were used for luciferase reporter assay.

Animals and other research organisms

Policy information about studies involving animals; ARRIVE guidelines recommended for reporting animal research, and Sex and Gender in Research

Laboratory animals	Four-week-old male BALB/c nude mice were purchased from the Shanghai Jiesijie Laboratory Animal Company and maintained under pathogen-free conditions. A549 cells were resuspended in 100 μL cold PBS and subcutaneously injected into the right flank of each nude mouse.
Wild animals	No wild animals involved in this study.
Reporting on sex	The findings were not applied to only one sex.
Field-collected samples	This study did not involve samples collected from field.
Ethics oversight	All animal studies were conducted in compliance with the policies of the animal ethics committee of Zhongshan Hospital, Fudan University (Approval No: B2022-180R).

Note that full information on the approval of the study protocol must also be provided in the manuscript.

Flow Cytometry

Plots

Confirm that:

- [x] The axis labels state the marker and fluorochrome used (e.g. CD4-FITC).
- [x] The axis scales are clearly visible. Include numbers along axes only for bottom left plot of group (a 'group' is an analysis of identical markers).
- [x] All plots are contour plots with outliers or pseudocolor plots.
- [x] A numerical value for number of cells or percentage (with statistics) is provided.

Methodology

Sample preparation	Lipid peroxidation test: Cells were seeded on 12-well plates and incubated overnight. The next day, treated cells were harvested by trypsinization and washed with phosphate-buffered saline (PBS, Beyotime). Next, the cells were suspended in a fresh medium containing 4 μM BODIPY 581/591 C11 dye (Thermo Fisher, USA) at 37°C in a humidified 5% CO2 atmosphere. After 30 min of incubation, the cells were washed with PBS, and the lipid peroxidation levels were assessed.
Instrument	BD Accuri C6
Software	The results were analyzed in FlowJo_v10 software.
Cell population abundance	At least 10000 cells were analyzed for each sample.
Gating strategy	Initial cell population gating (FSC-Area VS SSC-Area) was adopted to make sure debris was excluded, and then FSC-Area VS

FSC-Height was adopted to make sure only single cell was used for analysis.

☒ Tick this box to confirm that a figure exemplifying the gating strategy is provided in the Supplementary Information.
